# Strength Training Decreases Inflammation and Increases Cognition and Physical Fitness in Older Women with Cognitive Impairment

**DOI:** 10.3389/fphys.2017.00377

**Published:** 2017-06-12

**Authors:** Matheus U. Chupel, Fábio Direito, Guilherme E. Furtado, Luciéle G. Minuzzi, Filipa M. Pedrosa, Juan C. Colado, José P. Ferreira, Edith Filaire, Ana M. Teixeira

**Affiliations:** ^1^Faculty of Sport Science and Physical Education, Research Center for Sport and Physical Activity, University of CoimbraCoimbra, Portugal; ^2^Research Group in Prevention and Health in Exercise and Sport, University of ValenciaValencia, Spain; ^3^CIAMS, Université Paris-Sud, Université Paris-SaclayOrsay, France; ^4^CIAMS, Université d'OrléansOrléans, France; ^5^UMR 1019, INRA, Equipe ECREIN UNHClermont-Ferrand, France

**Keywords:** elastic band resistance training, older women, Interleukin-10, tumor necrosis factor-alpha, Interferon-gamma, C-reactive protein, cognition

## Abstract

**Introduction:** Cognitive impairment that affects older adults is commonly associated with an inflammatory imbalance, resulting in decreased physical fitness. Exercise has been pointed to mitigate immunosenescence and cognitive impairment associated with aging, while increase in physical fitness. However, few studies explored the relationship between changes in cytokine concentration and improvement on cognition due to elastic band strength training. The aim of this study was to investigate the effects of strength training on pro-and anti-inflammatory cytokines, hematological markers and physical fitness of older women with cognitive impairment.

**Methods:** Thirty-three women (82.7 ± 5.7 years old) participated in the study and were divided in two groups: strength exercise training group (ST; *n* = 16) and Control Group (CG; *n* = 17) and were evaluated before and after 28 weeks of the exercise program. The CG did not undergo any type of exercise programs. Data for IL-10, TNF-α, IFN-γ, C-Reactive Protein (CRP), white blood counts (WBC), red blood counts (RBC), Mini Mental State Examination (MMSE) and physical fitness tests were analyzed in both moments.

**Results:** IL-10 increased in the ST group without changes in CG. TNF-α and CRP increased in the control group while no changes were observed for IFN-γ in both groups. Strength training decreased leukocyte and lymphocyte counts and increase hemoglobin, mean cell volume and mean cell hemoglobin concentration. The MMSE score increased in strength training group but remained unchanged in the control group. A correlation between the variation of granulocyte counts and the MMSE scores was also observed within the total sample. An improvement in physical fitness was observed with strength training.

**Conclusion:** Resistance exercise promoted better anti-inflammatory balance and physical performance simultaneously with an increase in cognitive profile in older women with cognitive impairment.

## Introduction

Aging significantly affects immune function, including those process related to increased inflammation (Woods et al., 2012), physical function (Turner, 2016), and cognition (Chen et al., 2008). The deleterious effect of immunosenescence on cognitive profile has been widely studied (Au et al., 2016), and an increase in inflammation associated with a decrease in anti-inflammatory cytokines levels have been described as important contributors to cognitive impairment (CI) (Trollor et al., 2010). Inflammation can affect cognition by several mechanisms, either by the interaction of cytokines with specific receptors on peripheral afferent nerves that lead to neural transduction of signals in the central nervous system (CNS) activating microglia to produce cytokines (Quan and Banks, 2007), or by the transport of cytokines through the blood-brain barrier and subsequent interaction with the brain cells (Banks et al., 1998). This phenomenon is frequent in older people where higher levels of inflammatory markers like tumor necrosis factor alpha (TNF-α) and C-reactive protein (CRP) are found (Gorska-Ciebiada et al., 2015). TNF-α plays a key role on several immunological functions and it has been suggested that elevated levels of this protein are involved with risk for Alzheimer Disease (Tan et al., 2007; Phillips et al., 2014). CRP, a protein in the blood which is involved in the response to systemic inflammation, has been related to cognitive decline. Studies with older population samples show that higher levels of CRP are associated with cognitive decline (Yaffe et al., 2004), particularly in women (Watanabe et al., 2016). In fact, evidence in literature suggests that inflammation has a role in the pathogenesis of cognitive impairment and is more significantly observed in women than in men (Trollor et al., 2012). Also, the TNF-α/IL-10 ratio is a good index that represents the imbalance between the pro and anti-inflammatory systems in humans (Lira et al., 2009). Pre-conditioning exercise was recently shown to be able to ameliorate neurological injuries by enhancing anti-inflammatory responses in animal models (Chio et al., 2017), but further investigation regarding this association in humans is required. Moreover, the progressive alterations on hematopoiesis and blood rheology during aging can cause several disabilities like increased risk of anemia and poor physical performance (Simmonds et al., 2013). There is the understanding that changes in blood composition mediated by aging may result in risk of cognitive decline, but conclusions in this matter need to be better exploited, and the data remains controversial (Peters et al., 2008).

Strength and/or aerobic training have been pointed out as a practice that could mitigate the deleterious effects of immunosenescence (Beavers et al., 2010a). In older people, the importance of exercise has been assessed by its mediation in the treatment and/or prevention of inflammatory diseases that affect the cognitive profile. Evidence from regular aerobic training in this population showed that it caused a reduction of TNF-α plasma concentrations and an increase in the anti-inflammatory cytokine IL-10 (Santos et al., 2012). Regarding strength training (ST) previous evidence showed reduction in resting levels of TNF-α (Phillips et al., 2010) and CRP (Ogawa et al., 2010), contributing, in the long-term, to create an anti-inflammatory environment (Gleeson et al., 2011). Resistance exercise has numerous benefits for the elderly, since it can attenuate the loss of muscle mass during normal aging (Tseng et al., 1995), and increase muscle strength (Morganti et al., 1995). Besides, even in frail institutionalized subjects with more advanced ages (~90 years old), ST can increase functional mobility and muscle size (Fiatarone, 1990). In fact, relatively similar physiological responses are produced with ST in old and young individuals (Roth et al., 2001).

Because many traditional methods of strength training are not a viable option for some older persons, ST with elastic bands has been proposed as a safe, low cost and effective method to promote significant beneficial effects in these populations (Martins et al., 2013; Thiebaud et al., 2014), improving functional performance and muscle strength in healthy adults (de Oliveira et al., 2017) and lean body mass (Bittar et al., 2015). The use of elastic bands could be extremely convenient in older populations since their inexperience in strength training programs could be mitigated by a relatively simple and effective training that could promote short-term significant beneficial effects (Martins et al., 2013). Thus, the aim of this study was to investigate the effects of a chair-based elastic strength training (ST) on blood hematological markers and on pro-and anti-inflammatory cytokine balance in older women with cognitive impairment, as well as to assess if this type of exercise program could be used as a non-pharmacological tool in mitigating some of the aging effects described above. We hypothesized that 28 weeks of exercise practice would be able to create an anti-inflammatory balance and improve cognition in older women with cognitive impairment possibly associated with an improvement in physical fitness.

## Methods

This study was approved by the Ethical Committee of the Faculty of Sport Science and Physical Education—University of Coimbra [Ref.: CE/FCDEF-UC/000202013] and the protocols used are described in detail in the study protocol paper by Teixeira et al. (2016). All the participants completed and signed an informed consent form prior to their enrolment in the study. The entire study was conducted according to the Declaration of Helsinki to research in humans.

### Sample size

**Figure 1:**
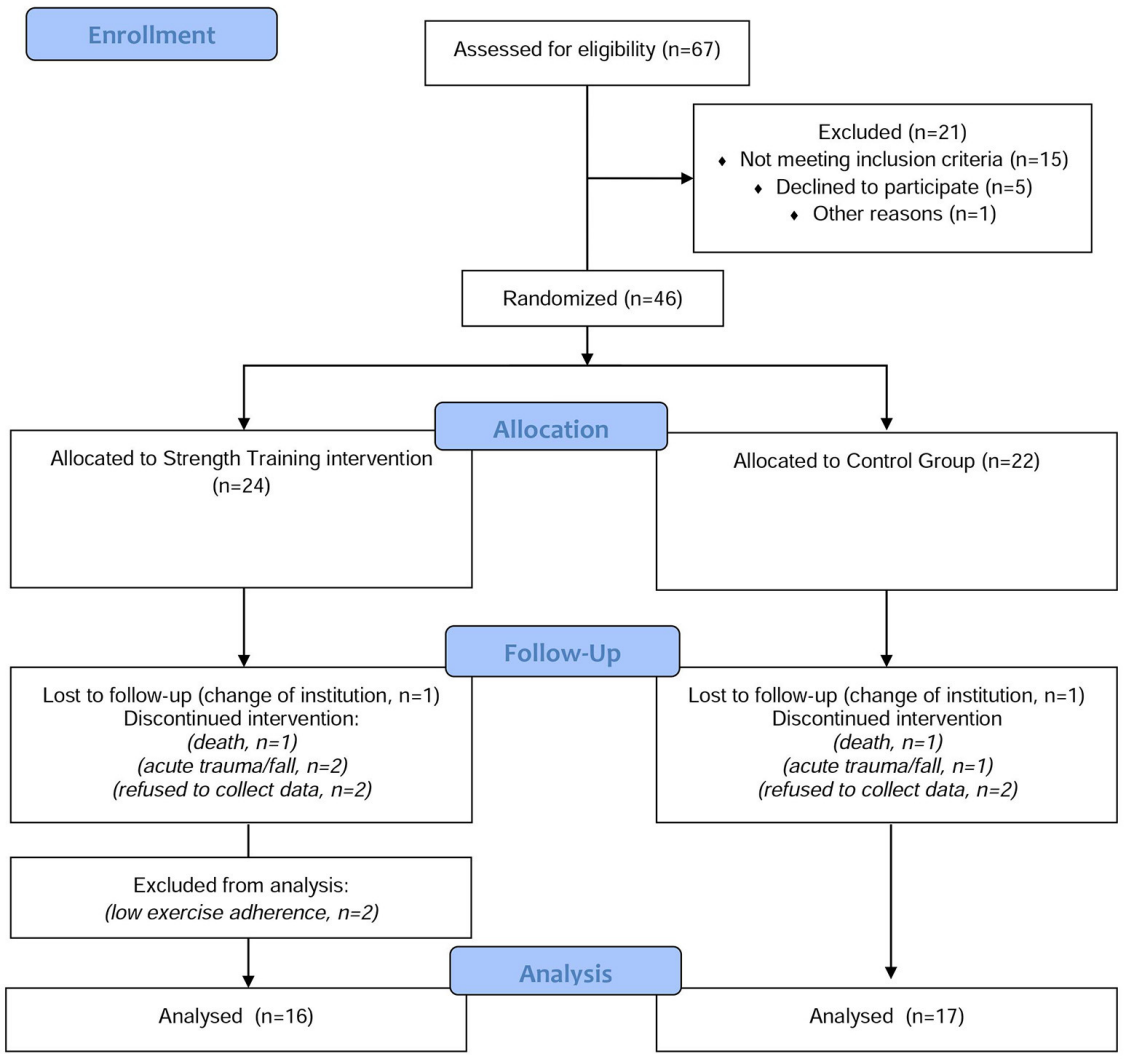
Flow chart of the study.

The estimated sample size was previously published by Teixeira et al. (2016). Briefly, alpha (type I error rate) was adjusted at 0.05 and power (type II error rate) at 0.85 using G^*^ Power (version 3.1.9.2). To provide enough information about the outcomes a required sample of 40 participants for both (intervention and control group) was estimated. Details of sample dropping-out are presented on flowchart (Figure 1).

### Eligibility criteria

The criteria used for inclusion in the study were: being a woman living in social and health care support center; age > 60 years; ability to practice moderate exercise without causing harm to themselves; designated as having moderate or mild cognitive impairment according to the Mini Mental State Examination criteria (MMSE) (Folstein et al., 1975) using education adjusted scores; willing to exercise for the entire study duration. After obtaining a signed consent term, data was collected regarding biosocial and global health, cognitive profile and general medical history. Based on this initial assessment, we excluded volunteers with the following history: recent head trauma, uncontrolled diabetes mellitus and hypertension; current chronic renal, liver or respiratory disorders; neurologic disorders or those with severe cognitive impairment (≤9 points on MMSE; Mungas, 1991); recent myocardial infarction (within the previous 6 months). We also excluded those individuals that were using hormone replacement therapy. The remaining subjects were allowed to continue using their usual medication, however, individuals who had unstable medical conditions, highlighted by the beginning of new medications within the data collection period, were also excluded.

### Measures

All baseline evaluations were performed during the morning, 2 weeks before the beginning of the strength training program. Evaluations occurred in separated days, beginning with the assessments of anthropometric measures, cognitive profile, and the hand-grip test. The following days were used to implement the physical fitness battery test. Collection of blood samples was done in a separate day by a registered nurse. The participants were warned 2 days in advance for the need to wear appropriate clothes and avoid ingestion of alcohol and caffeine in order to prepare for the physical tests. All data collection was done by a staff member and an assistant, and took place individually in a separate and quiet room (with adequate light and temperature) to maintain the participants privacy. To avoid interference of different criteria in data collection, each participant was always evaluated by the same staff members.

#### Global health

In order to determine the presence of comorbidities in the participants, all data regarding global health was obtained by interview the medical department of the social and health care institution and applying the Charlson Comorbidity Index (CCI) (Charlson et al., 1987). Based on 17 comorbid conditions it is reported to be able to predict 1 to 10-year mortality (Charlson et al., 1994).

#### Anthropometric measures

Body mass was determined using a portable scale (Seca, model 770, Germany) with a precision of 0.1 kilograms. A retractable glass fiber tape (Hoechstmass-Rollfix, Germany) was used to measure the waist circumference and the stature was determined using a portable stadiometer (SecaBodymeter, model 208, Germany) with 0.1 centimeters of precision.

#### Hand-grip test

The dynamometer (Lafayette, 78010, Indiana, USA) was used to measure the hand-grip strength test (HGT). The strength was measured in kilograms according to the standardized procedures described in Groningen Fitness Test for the Elderly (Lemmink et al., 2001). The subjects performed the test twice, in the standing position with the elbow at the side of the body. The handle size was adjusted and when ready, the participant squeezes the dynamometer with maximum isometric effort, which was maintained for 5 s. Thirty seconds of rest between measurements for both hands were given. The best value of each hand was computed and the higher value was used.

#### Physical fitness

To assess the physical fitness the Senior Fitness Test battery was used applying standardized procedures (Rikli and Jones, 2012). The tests stations were set in circuit-style to minimize fatigue and occurred in the following order: 8-Foot up and Go Test (8-FGT), “30's chair-and stand test” (30s-CS), “30's arm-curl test” (30s-AC) and the “2 min Step Test” (2m-STEP). The 8-FGT was used for evaluation of agility and dynamic balance, which assesses the time needed for the participant to get up from the chair, walk as quickly as possible around either side of a cone placed 2.44 m away and to sit back down in the chair. The lower body strength was determined with the 30s-CS, which measures the number of total stands completed in 30 s. The upper body strength was determined by the 30s-AC that measures the total number of arm curls executed in 30 s. Finally, the 2m-STEP is an alternative test for aerobic endurance that registers the number of times, within 2 min, that an individual can step in place. Participants performed 2–3 repetitions for each test to familiarize themselves with the tests. Each trial was applied with 2 min of relaxation between tests in a seated position. Before the start of the tests, participants engaged in 3 min of warm-up and stretching activities.

#### Assessment of cognitive profile

The assessment for cognitive impairment was performed using the Mini Mental State Examination, that gives information on five areas of cognition: orientation, immediate recall, attention and calculation, delayed recall, and language. The scores range from 1 to 30 (Folstein et al., 1975). The classification of cognitive profile in participants was categorized following the criteria previously described by Mungas (1991): severe cognitive impairment (values between 01 and 09); moderate cognitive impairment (between 10 and 18); mild cognitive impairment (between 19 and 24); normal cognitive profile (between 25 and 30). All questionnaires were applied by the same trained researchers at baseline and at the end of the exercise program in order to minimize for differences in procedures. Only those individuals classified with moderate and mild cognitive impairment (between 10 and 23 MMSE points) were enrolled in this study.

### Study design

A total of 33 (*n* = 33) women (age 82.7 ± 5.7 years) participated in the study (Body mass 66.8 ± 15.2 Kg; stature 150.6 ± 0.07 cm). They all lived in the same institution. The subjects were divided according to their groups, as: a chair-based elastic strength training group (ST; *n* = 16), and a Control Group (CG; *n* = 17) (Table 1). All subjects were evaluated before and after 28 weeks of the exercise program. The ST program started with 2 sessions/week during 8 weeks, with an increase to 3 sessions/week for 12 weeks, and returning to 2/week in the remaining 8 weeks of exercise, totalizing 28 weeks of intervention. The CG did not follow any type of exercise program.

**Table 1:** Participants characteristics at baseline.

	**Strength training (*n* = 16)**	**Control group (*n* = 17)**	***p***
	**Average (SD)**	**Average (SD)**	
Age (years)	83.50 (5.13)	82.12 (6.41)	0.382
Weight (Kg)	66.26 (16.35)	67.45 (14.57)	0.817
Height (cm)	150.4 (0.08)	150.8 (0.06)	0.581
Body Mass Index (kg/m^2^)	29.27 (7.10)	29.67 (5.98)	0.402
Charlson Comorbidity Index (score)	7.38 (2.39)	8.88 (2.78)	0.136
Hand-grip Test (kg)	12.75 (5.07)	12.70 (5.76)	0.901

*U-Mann Whitney Test; Data are Mean (SD)*.

### Exercise procedures

The development of the elastic band ST group was conducted by specialists in exercise prescription for elderly populations. Methods of design of exercise programs followed the recommendations of the American College of Sport Medicine/American Heart Association guidelines as a form of organization of the physical fitness program intervention (Nelson et al., 2007). The chair-based ST was used as an exercise class program developed using a specific number of sets, repetitions, and cadence execution and elastic bands with colors between yellow and red (TheraBand, Hygenic Corporation, Akron, OH, USA). The rest intervals between sets were done with the participants sitting in a chair. Two sessions of pre-intervention familiarization were conducted for participants to adapt at perceived exertion level and learn how to handle and the correct technique of the exercises. The program lasted 28-weeks and participants maintained a frequency of two times/week during the first 8 weeks of the program. The frequency increase to three times/weekly during the following 12 weeks and back to two times/week for the remaining 8 weeks. Each class had a maximum duration of 45 min divided into three parts with the following characteristics: [1] 5–10 min of warm-up (including six exercises for body mobilization and dynamic stretching); [2] 20–30 min of main part with muscle-strengthening activity (8–10 elastic-band exercises using the yellow and red colors levels of elastic bands); [3] 5–10 min of cool-down (specific exercises with easy stretching to promote cool down and relaxation) (Appendix I). During the first 14 weeks, the participants developed their ST using a yellow color elastic-band, to ensure that the exercise intensity was close to the desired one. In the remaining 14 weeks, the increase in the exercise intensity was induced by changing the elastic-band color to red. The exercise intensity was measured through the OMNI perceived exertion scale (PES) for strength exercise (Colado et al., 2012). The OMNI consists of an arbitrary scale ranging from 0 to 10 points, with identical intervals and regarding the quality of efforts: (0) extremely (1–2) easy, (3–5) somewhat easy, (6–7) somewhat-hard; (8) hard (9–10) extremely-hard). The intensity was kept with values of PSE between 6 and 8 levels. Heart rate monitors (Polar S810) were also used during the classes to record unexplained or abnormal alterations on heart rate and further control exercise intensity to avoid any cardiovascular risk.

### Exercise adherence

A total of 68 sessions were performed during 28 weeks. The exercise adherence to group classes was calculated individually through the total sum of participations. Entries were recorded in a checklist. When participants had two consecutive absences, they were contacted to return to the group classes. An adherence rate of 70% was established as the minimum for the ST exercise program.

### Blood sampling

Blood samples were collected during morning (from 10 a.m. to 11 a.m.) by venipuncture during rest by a registered nurse at baseline and after 28 weeks. All the subjects were encouraged not to engage in high physical efforts in the 24 h prior to the blood collection. Blood samples were drawn into vacutainer tubes containing EDTA for plasma and gel separator for serum analytics. After processing, plasma and serum they were allocated into eppendorfs and stored at −80°C until determination of the plasma concentrations of IL-10, TNF-α and IFN-γ, and the serum levels of CRP.

### Biochemical analysis

Inflammatory cytokines were analyzed using commercially available enzyme-linked immunosorbent assays according to the manufacturer's instructions from frozen plasma samples. Plasma levels of IL-10, TNF-α, and IFN-γ were measured by ELISA (Invitrogen, CA) following the manufacturer's instruction. The intra-assay coefficient of variability was 5.2% for IL-10, 4.1% for TNF-α, and 0.6% for IFN-γ. Serum CRP was determined using the Horiba Medical Pentra C200 analyser (Kyoto, Japan). A complete blood count analysis was performed using an automated hematology analyser Coulter AcT Diff (Beckman Coulter, USA).

### Statistical analysis

Statistical analysis was performed using SPSS (IBM, Statistics, version 22). Significance was set at *p* < 0.05. Descriptive statistics are presented as mean ± standard deviation. Since there was no normality distribution in cytokine levels in this sample the comparisons between groups at baseline were made using the Mann-Whitney *U*-test. Within-group comparisons between Pre and Post intervention were performed using the Wilcoxon's signed-rank test. Percent of change after intervention was calculated ([Post/Pre]-1) and presented for each variable. Correlations between parameters and between changes in variables were assessed according to Spearman's rank correlation coefficient. To report the strength of the exercise intervention the effect size (point-biserial correlation, *r*_pb_) was calculated and categorized as trivial (*r* ≤ 0.10), small (>0.10 to ≤ 0.25), medium (>0.25 to ≤ 0.36), and large effect (>0.36) (McGrath and Meyer, 2006).

## Results

All participants in the ST group completed at least 70% of classes during 28 weeks of the chair-based exercise program, and there were no observed adverse effects, injury or complications during this period related to the carried out intervention. However, some drop-out was observed among the groups during the 28 weeks of intervention. A total of 11 subjects who started the intervention were not reevaluated at follow-up [two subjects changed institution and stopped participating in the study; two subjects died, three participants had acute trauma (falls), and were no longer able to participate in the ST classes/data collection and four subjects refused data collection on follow-up]. All drop-outs experienced in the study were unrelated to the practice of ST. Only two participants that finished the study were excluded from the analysis due the low exercise adherence. Details of sample withdrawal are provided in Figure 1. Changes in cytokine levels, cognition and physical fitness in both groups are shown in Table 2. The WBC and RBC changes in ST and CG are presented in Table 3.

**Table 2:** Cytokine levels, MMSE and physical fitness tests scores in strength training and control groups.

	**Strength Training (*n* = 16)**					**Control group (*n* = 17)**				
	**Pre-intervention**	**Post-intervention**	***p***	**Percentage of change**	**Effect size (*r*)**	**Pre-intervention**	**Post-intervention**	***p***	**Percentage of change**	**Effect size (*r*)**
	**Average (SD)**	**Average (SD)**				**Average (SD)**	**Average (SD)**			
Interleukin-10 (pg/mL)	21.28 (12.55)	26.30 (12.77)	**0.026**	24	0.40	28.38 (12.03)	32.57 (9.24)	0.068	15	0.30
Tumor Necrosis Factor-α (pg/mL)	142.04 (65.24)	157.84 (58.41)	0.163	11	0.24	152.24 (76.53)	187.34 (67.75)	**0.006**	23	0.46
Interferon-y (pg/mL)	5.53 (3.48)	6.84 (4.63)	0.112	24	0.28	7.32 (5.04)	9.06 (3.06)	0.149	24	0.24
C-Reactive Protein (mg/L)	3.15 (3.41)	2.48 (2.02)^&^	0.469	−21	0.12	3.59 (3.1)	6.59 (3.0)	**0.001**	84	0.57
TNF/IL-10 ratio	7.60 (2.35)	6.38 (2.38)	0.148	−10	0.25	5.71 (1.85)	5.81 (1.4)	0.586	2	0.09
Mini Mental State Examination (pts)	15.69 (4.04)^#^	17.62 (3.61)	**0.017**	12	0.42	18.76 (4.09)	18.05 (5.3)	0.089	−4	0.29
Hand-grip Test (kg)	12.75 (5.07)	15.81 (2.07)^&^	**0.048**	24	0.35	12.70 (5.76)	13.0 (4.07)	0.667	2	0.07
8-FGT (seconds)	20.48 (9.3)	13.78 (5.1)^&^	**0.001**	−32	0.56	18.58 (6.6)	20.64 (9.0)	0.055	11	0.32
30s-CS (repetitions)	6.81 (2.5)	12.19 (2.7)^&^	**0.000**	79	0.62	6.71 (2.2)	6.76 (2.8)	0.964	1	0.00
30s-AC (repetitions)	11.19 (5.6)	14.88 (2.6)^&^	**0.013**	33	0.44	9.47 (4.0)	9.35 (3.6)	0.874	−1	0.02
2m-STEP (repetitions)	26.94 (15.4)	45.25 (12.9)^&^	**0.003**	68	0.53	30.0 (13.5)	25.94 (13.9)	**0.018**	−14	0.40

Data are Mean (SD, standard deviation); significance through Wilcoxon signed rank-test compared within groups to pre-post intervention. ^#^Different between groups at pre-intervention using U-Mann Whitney test;

& *Different between groups after 28 weeks (p < 0.05). Values of significance (p < 0.05) are highlighted in bold*.

**Table 3:** Hematological markers for the strength training and control groups.

	**Strength Training (*n* = 16)**					**Control group (*n* = 17)**				
	**Pre-intervention**	**Post-intervention**	***p***	**Percentage of change**	**Effect Size (*r*)**	**Pre-intervention**	**Post-intervention**	***p***	**Percentage of change**	**Effect Size (*r*)**
	**Average (SD)**	**Average (SD)**				**Average (SD)**	**Average (SD)**			
Leukocytes (× 10/uL)	7.05 (2.05)	6.48 (1.71)^&^	**0.004**	−8	0.51	7.83 (1.41)	8.16 (1.58)	0.215	4	0.21
Lymphocytes (× 10/uL)	2.11 (0.47)	1.75 (0.50)	**0.003**	−17	0.52	2.13 (0.63)	2.09 (0.51)	0.721	−2	0.06
Monocytes (× 10/uL)	0.35 (0.14)	0.36 (0.12)^&^	0.724	3	0.06	0.45 (0.19)	0.50 (0.11)	0.428	11	0.13
Granulocytes (× 10/uL)	4.59 (1.67)	4.38 (1.43)^&^	0.140	−5	0.26	5.27 (1.32)	5.61 (1.62)	0.201	6	0.22
Erytrocytes (× 10/dL)	4.49 (0.36)	4.37 (0.30)	0.132	−3	0.26	4.43 (0.40)	4.42 (0.44)	0.103	−3	0.27
Hemoglobin (g/dL)	12.16 (1.09)	12.80 (0.81)	**0.005**	5	0.50	12.01 (1.13)	12.15 (0.88)	0.338	1	0.16
Hematocrit (%)	39.36 (3.22)	39.43 (3.05)	0.609	0	0.09	38.84 (3.07)	38.03 (1.79)	0.407	−2	0.14
MCV (fL)	87.82 (5.61)	89.59 (5.31)	**0.001**	2	0.60	87.80 (6.69)	88.51 (5.89)	0.717	1	0.06
MCH (pg)	27.15 (2.08)	29.30 (1.86)	**0.000**	8	0.62	27.04 (2.66)	29.24 (1.75)	**0.000**	8	0.62
MCHC (g/dL)	30.90 (0.74)	32.70 (0.39)	**0.000**	6	0.62	30.78 (0.98)	32.57 (0.57)	**0.000**	6	0.62

*Data are Mean (SD, standard deviation); significance through Wilcoxon signed rank-test compared within groups to pre-post intervention*.

& *Different between groups after 28 weeks (p < 0.05). Values of significance (p < 0.05) are highlighted in bold. MCV, mean cell volume; MCH, mean corpuscular hemoglobin; MCHC, mean corpuscular hemoglobin concentration*.

### MMSE score

Although, the average levels of the MMSE scores for the control group were higher than those for the strength training group before the intervention (*p* < 0.05), both groups were in the same category of cognition (moderate cognitive impairment). A significant increase and large effect size were observed on MMSE score after 28 weeks of ST (*p* = 0.01; Δ = 12%; *r*_pb_ = 0.42), while no change was observed in the CG for the same period (*p* = 0.08; Δ = −4%; *r*_pb_ = 0.29).

### Cytokine concentrations

**Figure 2:**
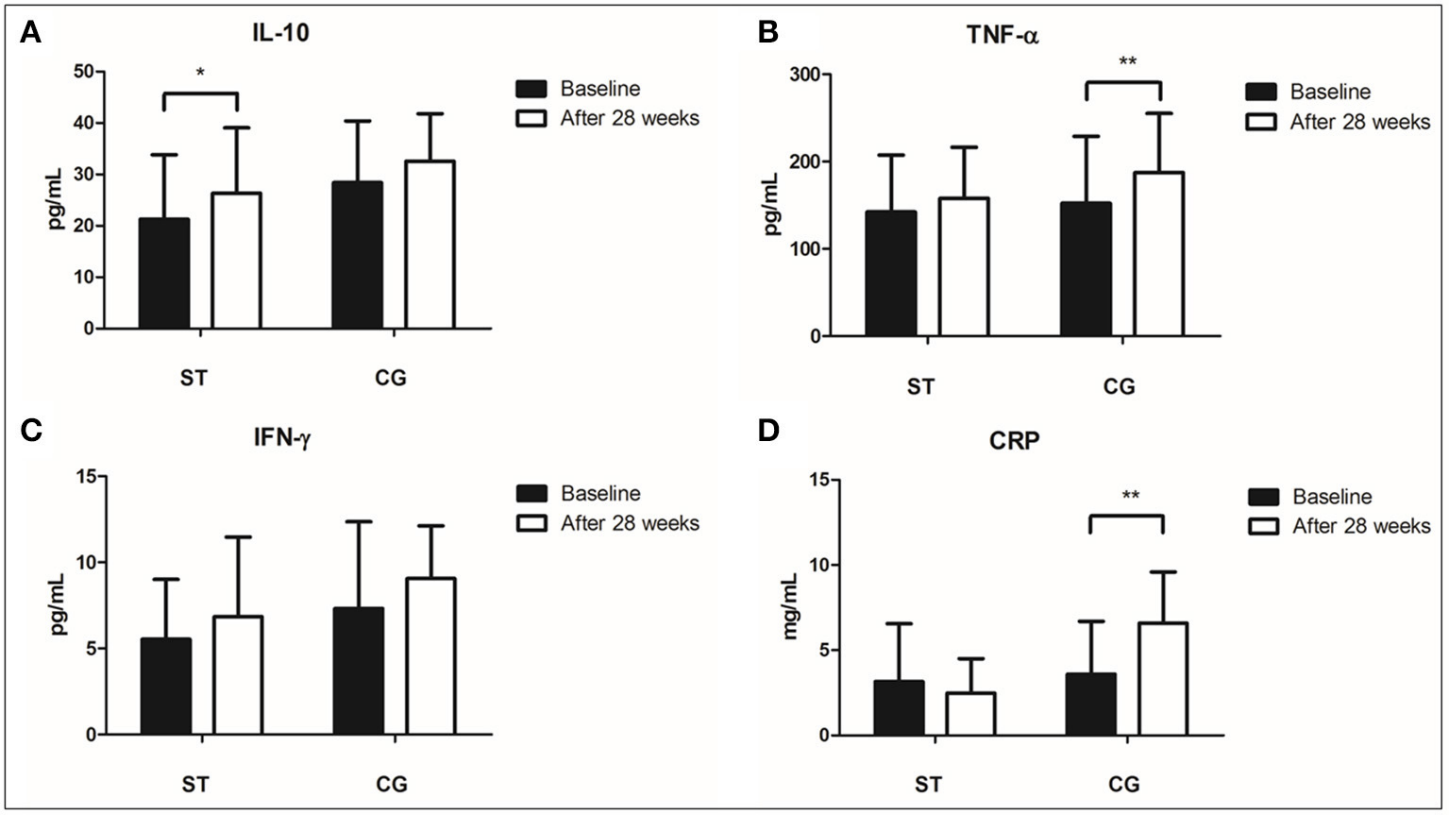
Differences in Cytokine concentrations from baseline to the end of the intervention for the exercising and control groups. Data are presented as mean with standard deviation bars. ^*^p < 0.05; ^**^p < 0.01. **(A)** Interleukin 10; **(B)** Tumor Necrosis Factor-alpha, **(C)** Interferon gamma; **(D)** C-Reactive Protein; Concentrations before (black columns = baseline) and after (white columns = 28 weeks of intervention), in Strength Training group (ST) and Control Group (CG).

After intervention, IL-10 concentrations increased significantly in the ST group (*p* = 0.02; *r*_pb_ = 0.4) and no significant changes were observed for the CG. Despite a subtle alteration in the mean levels of circulating INF-γ, this parameter remained unchanged after intervention both in the ST and Control groups (*p* = 0.112 and *p* = 0.149, respectively). At the same time, concentrations of TNF-α increased significantly only in the CG (*p* = 0.006; Δ = 23%; *r*_pb_ = 0.46). CRP concentrations did not change in the ST group, however, a significant increase was observed in CG (*p* = 0.001; *r*_pb_ = 0.57). Meanwhile, the TNF-α/IL-10 ratio showed a subtle decrease only in ST group (Δ% = −10%;*r*_pb_ = 0.25), but with no statistical significance (*p* = 0.148). Such tendency was not observed in the CG (Figure 2).

### WBC

**Figure 3:**
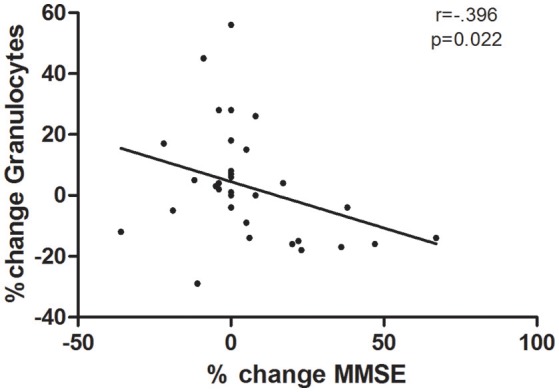
Correlation between changes in Granulocytes counts and MMSE score calculated using Spearman's rank correlations between percentage changes ([post-value/pre-value] −1) in the total sample.

After 28 weeks the total number of leukocytes and lymphocytes decrease in the ST (Δ = −8%, *r*_pb_ = 0.51 and Δ = −17%, *r*_pb_ = 0.52, respectively) while no changes were observed in the CG. Although there were no significant changes in the granulocyte count in both groups between pre-and post-values, differences in granulocytes were observed between groups after the intervention (*p* = 0.04). The correlation presented in Figure 3 shows that while the variation of granulocyte count decreased there was an increase in the MMSE score observed within the total sample (*r* = 0.396, *p* = 0.02).

### RBC

Results indicate an increased hemoglobin (*p* = 0.005, *r*_pb_ = 0.50), mean cell volume (*p* = 0.001, *r*_pb_ = 0.60), mean cell hemoglobin (*p* < 0.001, *r*_pb_ = 0.62), and mean cell hemoglobin concentration (*p* < 0.001, *r*_pb_ = 0.62) in the ST group after the exercise program. There were no significant changes in these variables for the CG, except the increase of mean cell hemoglobin (*p* < 0.001, *r*_pb_ = 0.62) and mean cell hemoglobin concentration (*p* < 0.001, *r*_pb_ = 0.62).

### Physical fitness

**Figure 4:**
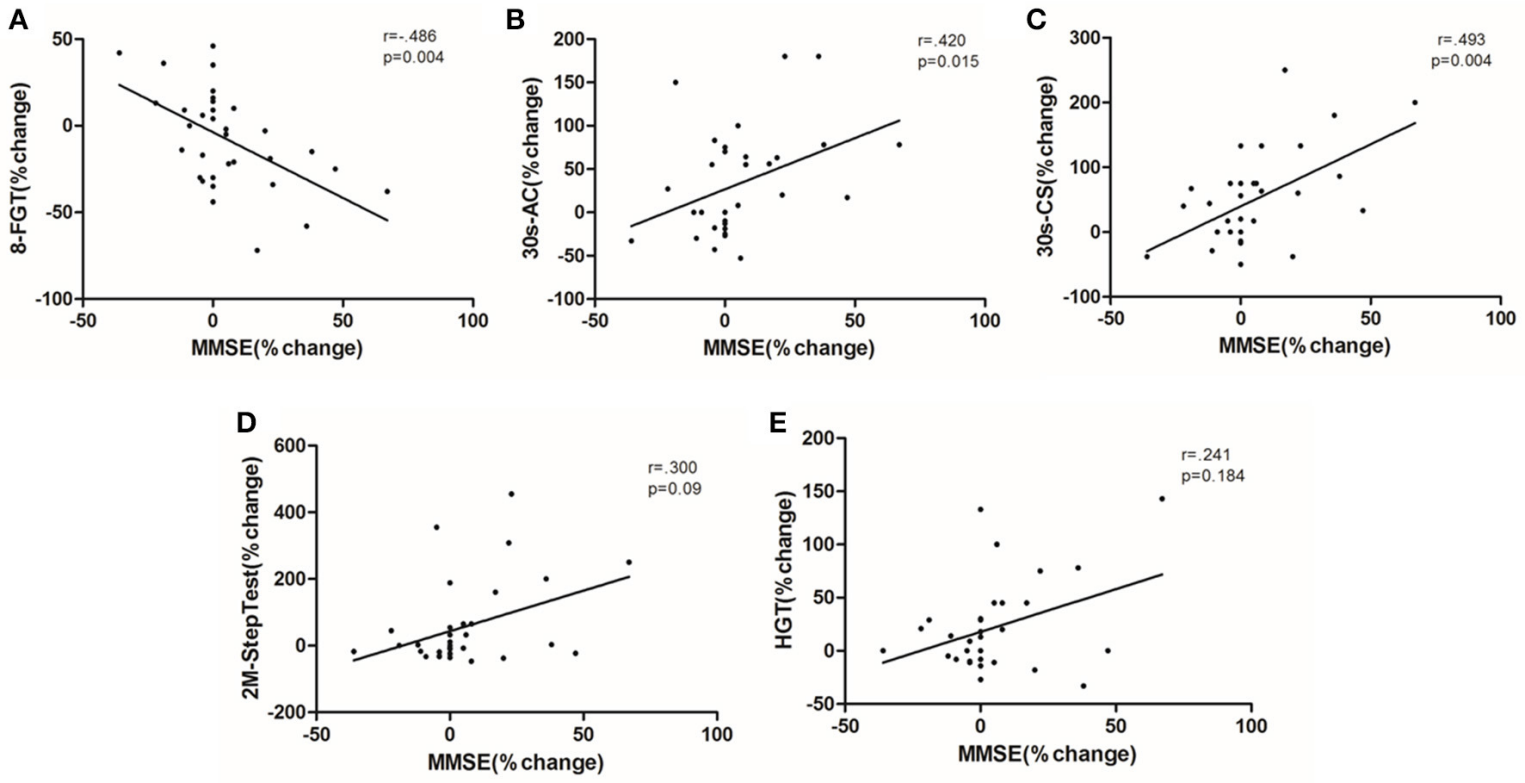
Correlations between changes in the Physical Fitness tests and the MMSE score calculated using the Spearman's rank correlations between percentage changes ([post-value/pre-value] −1) in the total sample. **(A)** 8-foot-up and go test; **(B)** 30-s arm curl test; **(C)** 30-s chair-and stand test; **(D)** 2-min step test; **(E)** hand-grip test.

A decrease in time for the 8-FGT test (*p* = 0.001), as well as an increase in the number of repetitions for the 30s-CS, 30s-AC and 2m-STEP tests (*p* < 0.001, *p* = 0.013, and *p* = 0.003, respectively) were observed in the ST group with a large magnitude effect (Table 2). No significant changes were found for the CG in any of the physical fitness variables tested, except a decrease for the 2m-STEP test (*p* = 0.01). Changes in MMSE scores correlated significantly with changes in the 8-FGT (*r* = 0.004), 30s-CS (*r* = 0.015), and 30s-AC (*r* = 0.004) tests (Figure 4).

## Discussion

The aims of this study were to investigate the effects of chair-based elastic-band strength training on blood hematological markers and on inflammatory balance in older women with cognitive impairment, as well as to assess if this type of exercise program can be used as a non-pharmacological tool in mitigating some of the aging effects described earlier.

The main findings of this study were that the 28 weeks of elastic-band strength training program could increase functional fitness and anti-inflammatory cytokine concentrations together with the attenuation of inflammation and improvement of global cognition in institutionalized older women with cognitive impairment. At the same time, we saw that the variation (decrease) of granulocytes numbers was correlated with an increase on cognition (Figure 3). So, these non-traditional methods based on elastic bands are very practical because they are low cost, accessible, and effective, and the qualified personnel could readily use these materials to develop and easily supervise such programs (Flandez et al., 2016).

The regular levels of physical activity and total energy expenditure in institutionalized elderly populations are usually lower than those who live independently (Król-Zielińska et al., 2011). In addition, the presence of cognitive impairment reduces levels of daily physical activity in older adults compared to those with a better cognitive profile (Simpson et al., 2012; Woods et al., 2012). Exercise has a very important role in increasing the energy expenditure and regulating the immune system (Król-Zielińska et al., 2011). Indeed, exercise practice has been used as non-pharmacological therapy on a phenomenon called immunosenescence, a process by which aging negatively affects the immune function. Evidence in the literature already shows that, even in older individuals, immunological adaptations that improve the anti-inflammatory profile and reduce inflammatory cytokine levels can be achieved through regular exercise practice (Gleeson et al., 2011; Simpson et al., 2012). There is however a lack of information on the cognitive profile of institutionalized old participants involved in longitudinal studies with exercise and its effect on inflammatory indices. In our study both groups were classified within the same category of cognitive profile (moderate cognitive impairment) (Mungas, 1991). In studies reporting interventions longer than 16 weeks, the effects of exercise training as a mean to reverse immunosenescence in previously sedentary older persons are still controversial. On the other hand, intervention studies with more than 6 months of duration seem to be more effective (Kohut and Senchina, 2004). In fact, our results showed that 28 weeks (i.e., 7 months) of strength exercise ameliorated the anti-inflammatory balance by increasing anti-inflammatory cytokine concentrations and decreasing total leukocyte and lymphocyte numbers.

Most of the studies that evaluated the effects of physical exercise (with more than 24 weeks of intervention) on cognition had an average sample age smaller than that of our study (Etnier et al., 2006). Our results showed that strength training was able to improve performance on the MMSE tasks and to increase the global cognition levels of older women with CI, which did not occur in those from the control group. In accordance with the above mentioned review, it seems that the increment of physical activity levels is an important factor in improving cognition in older women. Our results also show correlations between improvement in the MMSE scores and physical fitness tests (Figure 4) corroborating other studies that report that exercise can attenuate cognitive decline over time (Muscari et al., 2010), or even improve it after long-term interventions (Kwak et al., 2008; Hars et al., 2014). These correlations can be explained recognizing that physical fitness tests involves, in addition to physical requirements, some attention, concentration and comprehension of skills, and these areas are sensitive to the cognitive test used. Since the elderly have their cognition improved, performance on the test can be improved as well. Although the increase observed in cognitive profile in ST group did not change the characterization of moderate cognitive impairment, it was interesting to observe that this elevation (+12%) occurred only in the exercise group. Recently, it was shown in an animal model that pre-conditioning exercise protected the neurons against inflammation by improving the anti-inflammatory HSP70/NF-κB/IL-6/synapsin I axis (Chio et al., 2017). This mechanism may contribute to the attenuation of cognitive loss associated with “*inflammaging*” and, in fact, our results showed that increases in IL-10 and maintenance of TNF-α levels resulted in a better inflammatory balance in the elderly who performed the ST, which in turn may have contributed to the maintenance of cognition in this group.

IL-10 is a key anti-inflammatory cytokine that acts on the inhibition of systemic inflammation and is also pointed to play an important role on the inhibition of TNF-α production (Saraiva and O'Garra, 2010) as corroborated by the correlation between the changes in IL-10 and TNF-α levels found in our study. The increased concentrations of IL-10 found in our exercise group corroborate other findings involving cytokines and its response to exercise programs in older persons (Rodriguez-Miguelez et al., 2014). This mechanism is supported by the immunoregulatory effect of exercise practice that increases IL-10 and attenuates TNF-α levels (Moldoveanu et al., 2001). The increased physical activity during 28 weeks can be constructed as a central way to create an anti-inflammatory environment in older people, since an increase in IL-10 levels occurred conjunctly with a slight decrease in the TNF-α/IL-10 ratio that was not seen for the CG (Table 2).

Literature review suggest that older people who are physically active present less systemic inflammation compared to those who do not practice any exercise (Geffken et al., 2001; Bruunsgaard, 2005; Beavers et al., 2010a). However, it must be considered that most of the studies are correlative and not causal, which makes difficult to extrapolate data regarding the role of physical exercise programs offered to older people during long periods and its effects on inflammatory indices. A significant increase in TNF-α levels was observed in the CG after the 28 weeks period. Considering that circulating pro-inflammatory TNF-α and CRP can contribute to neuronal damage and act on cognitive dysfunction, low concentrations of these inflammatory markers are fundamental for the maintenance of a good anti-inflammatory environment in the brain. The group who performed strength training prevented this increase over time, which shows that this type of exercise may play an important role in suppressing TNF-α effects probably due to the increase in IL-10 secretion. Similarly to others studies, our results found no reductions in TNF-α concentrations after the exercise program (Rall et al., 1996; Beavers et al., 2010b). More recently, a multimodal physical exercise with aerobic and strength components lasting 16 weeks was able to reduce TNF-α levels and improved cognition in older people (Nascimento et al., 2014). Even so, the differences in sample average ages and type of exercise between studies should be taken into consideration. It must be considered that our study (carried out in a nursing home care center) exerts a greater control of the variables involved, in contrast with others studies done with older people performing home-based exercise programs (Ohta et al., 2012; Nishida et al., 2015). Resistance exercise can attenuate the age-associated muscle wasting by suppressing the skeletal muscle expression of TNF-α in old frail men and women (Greiwe et al., 2001). Methodological differences make it difficult to compare changes in TNF-α concentrations over time, since some studies do not have a control group (Santos et al., 2012) or were done with young individuals (Ploeger et al., 2009; Della Gatta et al., 2014).

The TNF-α/IL-10 ratio can be indicative of the balance between the pro and anti-inflammatory status (Lira et al., 2009). In our study, a medium effect size for the decrease in the TNF-α/IL-10 ratio was observed only in the ST group (−10%, *r*_pb_ = 0.25), which ties in with the increased in IL-10 and maintenance of TNF-α levels observed after 28 weeks of exercise. Indeed, these changes were not observed (the TNF-α/IL-10 ratio did not change) in the CG where a rise in pro inflammatory markers was observed, with CRP levels increasing after 28 weeks. Some studies show that individuals who rarely were enrolled in exercise programs had higher CRP levels compared to those that practiced strenuous physical activity (Albert et al., 2004), which supports the idea that inflammatory markers such as CRP are more sensitive to physical activity than just age *per se* (Giannopoulou et al., 2005). Previous evidence with older subjects does suggest that CRP levels decrease after training (Muscari et al., 2010; Rodriguez-Miguelez et al., 2014). CRP values have already been pointed out as possible predictors of dementia in the elderly, especially in those with pre-existing cardiovascular disease (Hsu et al., 2017; Weinstein et al., 2017). Although there is no correlation with CRP and cognitive profile in our study, it is interesting to observe that the increase of this biomarker occurred only in the control group, simultaneously with a slight decrease in cognition. The practice of ST using elastic bands may have a public-health relevance for older women, since a prospective study suggested that an increase of 1.02 mg/L of CRP was correlated with 35% of increase risk to develop colon cancer (Erlinger, 2004). Some specific pathways can induce elevation of CRP in serum, however, looking at our results it is possible that this elevation may be mediated by TNF-α, since increases in cytokines such as TNF-α and IL-6 stimulate the hepatic release of CRP (Campbell et al., 2009).

Despite a subtle increase, IFN-γ plasma levels did not significantly change in both groups. The precise role of IFN-γ in health is complex, since it has an important role in the control of inflammation, ranging from the migration of leukocytes to the activation of its effector function (Rauch et al., 2013). It also stimulates the production of reactive oxygen species and inflammatory cytokines (Marchi, 2014). A recent study showed an association between reduced IFN-γ and increased HDL-cholesterol levels in elderly women enrolled in an exercise program (Nishida et al., 2015), which pointed to the beneficial effect of physical training on lipid profile associated with the reduction of circulating IFN-γ. On the other hand, immunosenescence is characterized by a decrease in IFN-γ probably due to a shift from Th1 to Th2 cells (Bruunsgaard and Pedersen, 2000).

When looking at the changes observed for WBC and RBC, these could be explained by several mechanisms through which exercise can act on their values. The leukocyte traffic through lymphoid organs and blood may be altered due to exercise practice, since aerobic training has previously been shown to decrease total WBC in sedentary post-menopausal women (Johannsen et al., 2012). Excluding the effect of acute infection, our results showed that elastic band ST decreased the total number of leukocytes and lymphocytes in older women with a large magnitude of effect (*r*_pb_ = 0.51 and *r*_pb_ = 0.52 respectively). In fact, individuals who exercised throughout life have WBC levels lower than previously sedentary older people (de Gonzalo-Calvo et al., 2012). Also, in our study, a decrease in granulocyte counts was associated with the increase in cognition after 28 weeks of exercise. A relationship between high WBC counts and low cognition has previously been demonstrated (Kao et al., 2011; Cohen-Manheim et al., 2015), supporting the idea that vascular inflammation induced by aging can affect cognition. Although not conclusive, these effects support the evidence that physical exercise decreases leukocyte levels associated with the improvement of global health in older people. Our study also showed significant changes in hemoglobin, mean cell volume, mean corposcular hemoglobin and mean corpuscular hemoglobin concentration in the exercising group, which seems to demonstrate an effect on blood rheology, namely with an improvement in the mechanisms that enhance oxygen transport. It is remarkable to observe that the initial health condition of the elderly seems to affect the response to the exercise, since a previous study with healthy older women showed no differences on those variables after 6 months of resistance training (Bobeuf et al., 2009). Low hemoglobin concentration can decrease global cognition by several possible mechanisms, including negative influence on cardiovascular function (Inzitari et al., 2008) and poor neuroprotection in elderly with chronic kidney disease (Hong et al., 2013). Although promising, the implications of exercise training on hematopoiesis still requires further investigation in this population.

In conclusion, our study suggests that 28 weeks of chair based strength exercises were able to increase the anti-inflammatory balance in older women simultaneously with an increase on cognitive profile and better physical performance even in the presence of cognitive impairment.

## Ethics statement

This study was carried out in accordance with the Portuguese Resolution (ART 176 4st; Law no. 12/2005, 1st series) on ethics in research with humans. All subjects gave written informed consent in accordance with the Declaration of Helsinki. The protocol was approved by the Faculty of Sport Sciences and Physical Education Ethical Committee – University of Coimbra (number reference: CE/FCDEF-UC/000202013), and is an integral part of the research project entitled “PRO-HMECSI: Hormonal mediation of exercise on cognition, stress and immunity”.

## Author contributions

MC, GF, and LM participated in data collection, analysis and wrote the manuscript. FD and FP participated in data collection. JC and EF made contributions in their fields. JF and AT coordinated the research.

### Conflict of interest statement

The authors declare that the research was conducted in the absence of any commercial or financial relationships that could be construed as a potential conflict of interest.

## Appendix I

**Table:**

**Phase 1 (week 1–14)**			
**Warm-up (5–10 min)**	**Main part of the ST (20–30 min)**	**PSE**	**Cool-down (5–10 min)**
General body mobilization and dynamic stretching chair-based exercises; mobilization of the shoulder girdle; familiarization with the elastic bands (moves the elastic upward; stretch the elastic until the beginning of the tension point); dynamic chest (hug movement); pelvis mobility with leg abduction or knee flexion; dynamic squat front arm in the chair; simple spine twist (chair stand)	Muscle-Strengthening Activity: 8–10 exercises using the first level (yellow) of elastic-band Thera-band.	3–4	Unilateral quadriceps stretch; hamstring stretch; calf stretch in the wall; spine twist stretch (chair/stand); sideways static stretch (chair/stand); upper back stretch (chair/stand); chest stretch (chair/stand); unilateral triceps stretch
	Sets (1–2); Repetitions (10–12); cadence (2:3), rest (45″).		
	Exercises^*^: front squat (stand or chair); unilateral hip flexion with chair; bench over row (with flexion); chest press (stand or chair); standing reverse fly; spine twist extension arm (oblique's); shoulder press/twist arm front position; frontal total raiser; biceps arm curl (stand or chair); overhead triceps extension.		
	**Phase 2 (week 15–28)**		
	Muscle-Strengthening Activity: 8–10 exercises using the second level (red) of elastic-band Thera-band.	6–7	
	The progress of ST was partly based on change of elastic-band used (yellow to red) which create more tension during the concentric and eccentric movement. Additionally, were used more complex progressions (example: 2–3 sets × 10 repetitions of front Squat in the chair + frontal raiser).		

* *Unilateral exercise was done one set on each body side (left/right)*.
